# Suicide by sharp force associated with major self-mutilation and self-cannibalism

**DOI:** 10.1007/s12024-023-00674-7

**Published:** 2023-07-14

**Authors:** Silvia Farkašová Iannaccone, Alžbeta Ginelliová, Daniel Farkaš, Dorota Sopková

**Affiliations:** 1 Department of Forensic Medicine, Faculty of Medicine, Pavol Jozef Šafárik University in Košice, Trieda SNP 1, 040 11 Košice, Slovakia; 2 Medico-Legal and Pathological-Anatomical Department of Health Care Surveillance Authority, Ipeľská 1, Košice, 043 74 Slovakia

**Keywords:** Suicide, Sharp force injuries, Self-mutilation, Self-cannibalism, Forensic autopsy

## Abstract

Self-stabbing and self-cutting represents an uncommon method of suicide. We present a case of a 30-year-old man who was found dead in the forest. The body was naked and showed multiple cut and stab wounds on different parts of the body (face, neck, chest, abdomen, and extremities). A single-edged kitchen knife was found approximately 20 m from the body. Parts of both ears, the fifth toe of the right foot, and the scrotum were cut off. At the autopsy, two of the severed body parts—the toe and the part of the left ear—were found in the stomach. The cause of death was asphyxiation due to blood aspiration resulting from a cut throat injury. A police investigation uncovered a history of substance abuse and two previous suicidal attempts using a knife. Upon complex analysis of all the evidence, the manner of death was ruled a suicide, which was preceded by actions of major self-mutilation and self-cannibalism, both considered rare behavioral patterns.

## Case report

**Fig. 1:**
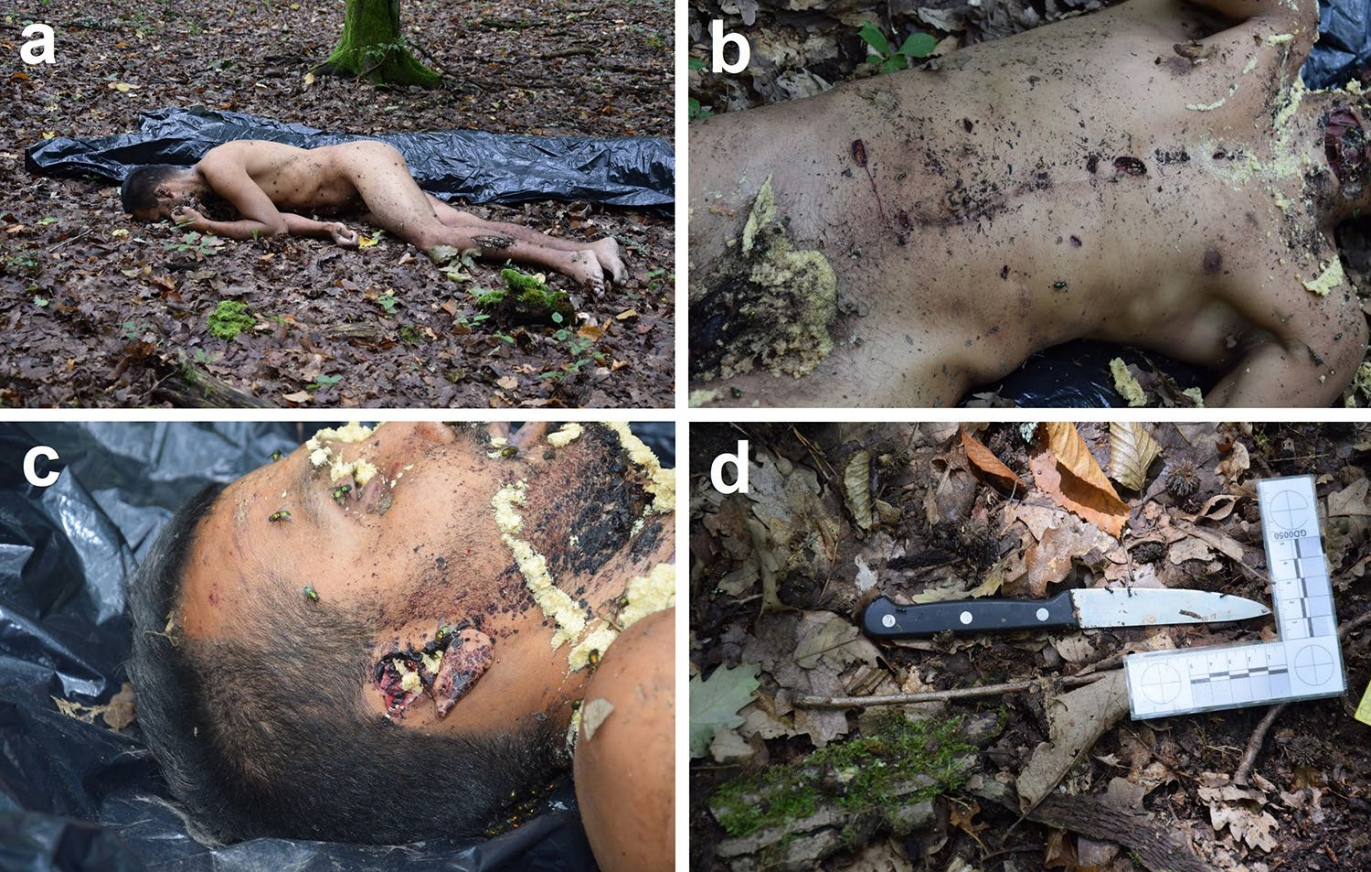
The photographs taken at the place where the body was found: the body of the deceased lying face down on the ground (**a**), the anterior aspects of the body showing multiple sharp force injuries and the presence of fly eggs and maggots (**b**), the right aspects of the head showing part of the right ear being cut off (**c**), and the kitchen knife found in the forest (**d**)

A 30-year-old man was discovered dead in the forest adjacent to a village during the summertime at about 8 a.m. The body was naked, lying on its right side with the face touching the ground (Fig. 1a). Initial inspection revealed multiple cut and stab wounds (Fig. 1b). Parts of both ears, the fifth toe of the right foot, and the scrotum were cut off (Fig. 1c). There were no severed body parts discovered nearby or farther into the forest. The clothes were also not found. The face, chest, external genitals, and some of the wounds were covered by fly eggs and maggots. A single-edged kitchen knife with a 10-cm long blade was discovered on the ground approximately 20 m from the body (Fig. 1d).

**Fig. 2:**
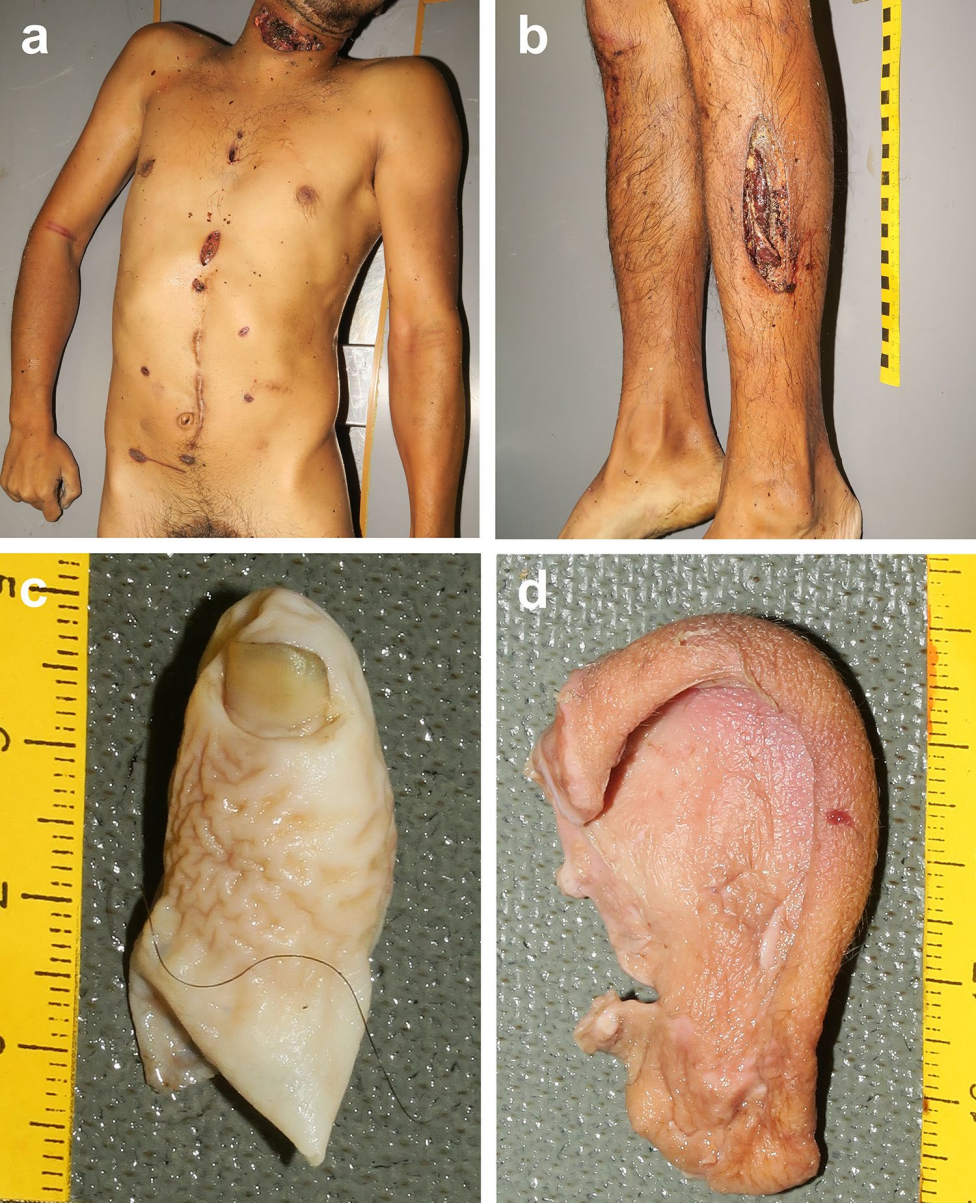
The photographs taken at autopsy: anterior aspects of the body showing multiple sharp force injuries and scars (**a**), a longitudinal cut wound on the left lower leg (**b**), the fifth toe of the right foot (**c**), and part of the left ear (**d**) found in the stomach

**Fig. 3:**
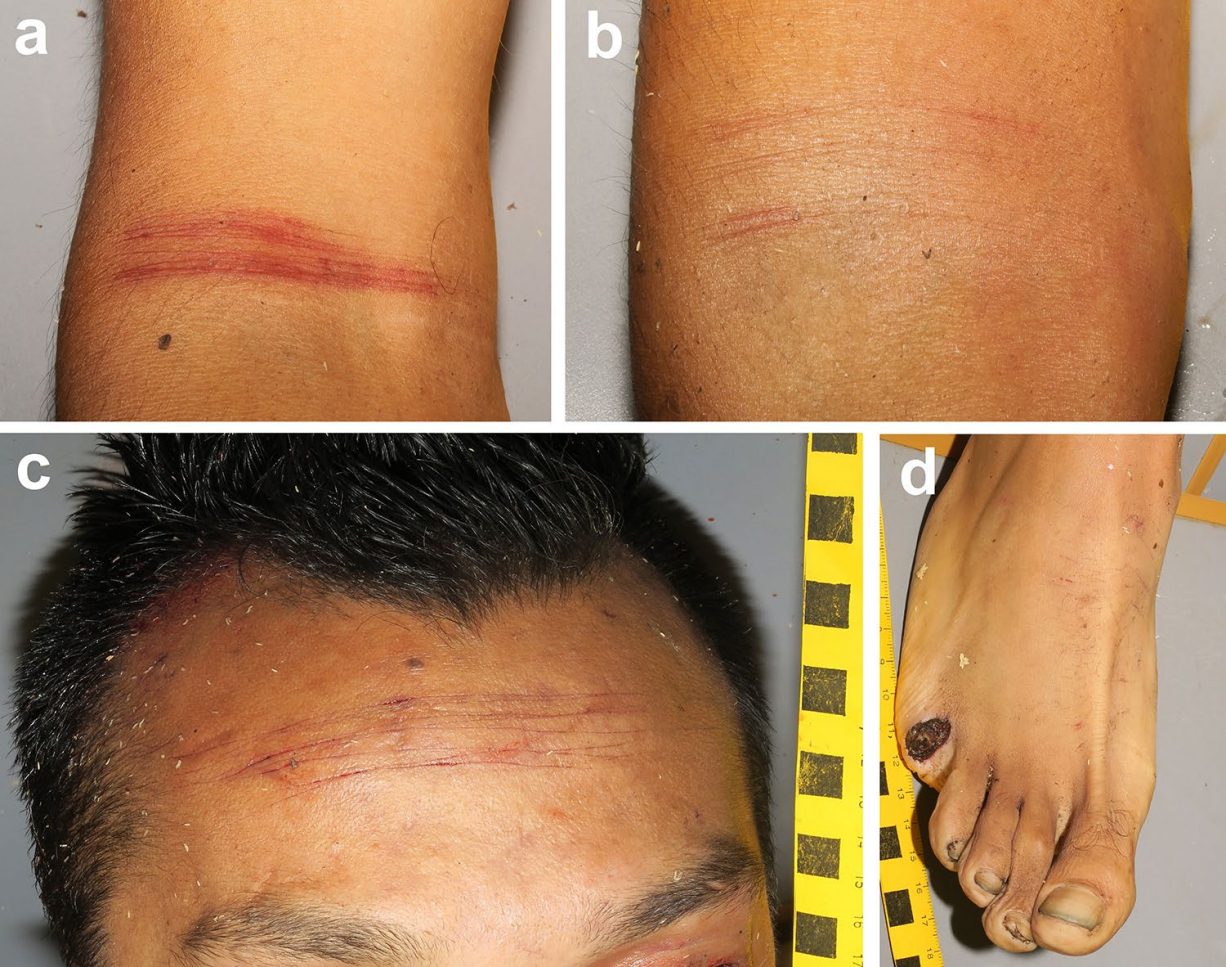
The photographs taken at the autopsy: multiple superficial cut wounds on the right elbow (**a**), left elbow (**b**), forehead (**c**), and right foot with the fifth toe being cut off (**d**)

The autopsy was performed the next day after the body was found. The body measured 166 cm and weighed 55 kg. External examination of the body revealed 1 deep transverse cut wound on the neck (10 cm long) and on the left side of the mouth (2 cm long), and 1 longitudinal cut wound on the left lower leg (13 cm long) (Fig. 2b). The palms of both hands, dorsum of the left foot, and plantar surface of the right foot presented with a few differently oriented isolated superficial cut wounds not longer than 2 cm; 1 transverse superficial cut wound (5 cm long) was present on the abdomen. Hesitation marks in the form of superficial cuts oriented parallel to each other were found on both elbows (Fig. 3a, b), the forehead (Fig. 3c), and the dorsum of the right foot (Fig. 3d). There were multiple stab wounds affecting only subcutaneous tissue on the neck (2×), chest (3×) (Fig. 2a), and abdomen (4×) (Fig. 2a), and a single stab wound on the right tight, left lower leg, and dorsum of the left foot. In addition to these sharp force injuries, there were small abrasions on the face, chest, back, left arm, both knees, and right lower leg, as well as a laceration under the right eye and on the right knee. Scars were present on the chest, abdomen (Fig. 2a), right elbow, and right wrist. A transection of the trachea and right internal jugular vein was discovered as a result of a cut wound on the neck. There was blood present in the trachea and bronchi. Both lungs were hyperinflated, and signs of blood aspiration were visible on the cut surface. Intact part of the left ear and a toe were found in the stomach (Fig. 2c, d). Other autopsy findings included brain edema, petechial hemorrhages in the scalp and under the pleura, adhesions in the left thoracic cavity and abdominal cavity, and signs of intestinal surgery performed in the past. Toxicological analyses of peripheral venous blood did not detect alcohol or any psychoactive substances. The cause of death was asphyxiation due to blood aspiration.

During the police investigation, the parents of the deceased stated that he was living with them in the village near the forest where his body was found. He has been abusing synthetic cannabinoids known as Herba since he was 16 years old, but he has not taken drugs for the past 3 years. Two years prior to his death, he started hearing voices that commanded him to kill himself. At that time, he tried to commit suicide by stabbing himself in the chest. Following his suicidal attempt, he was diagnosed with psychotic disorder with delusions, and he started psychiatric treatment. The same year, he made another suicidal attempt, using a kitchen knife to cut his neck and abdomen while damaging multiple abdominal organs. Since then, his psychiatric treatment has changed and become more frequent. He was taking olanzapine and mirtazapine. People in the village last saw him around 3 p.m. the day before his body was discovered in the forest. He was clothed then, and his behavior seemed normal. The parents recognized the knife found in the forest as being from their kitchen. DNA analysis of the blood collected from the knife proved that the blood was a match with the blood of the deceased collected during the autopsy. No other DNA profile was detected. There were no fingerprints on the knife. The deceased was left-handed.

## Discussion

Self-cutting or self-stabbing is not a common method of suicide, while both of these sharp force injuries are equally represented [1–4]. Differentiation between suicide and homicide is often based on several features, including the number, location, and direction of the wounds, the presence of hesitation marks, and the absence of clothing injuries. An accurate determination of the manner of death cannot be made solely based on the autopsy. The findings must be interpreted within the context of information obtained at the scene, death circumstances, victim’s handedness, and medical history [5–7]. The most frequent sites of self-inflicted cut wounds are the neck, elbows, and wrists. In the case of a stab wound, it is the left side of the chest, which is believed to be the anatomical position of the heart, but also the upper abdomen, for the lack of obstacles, such as ribs, preventing entry into the vital organ [4, 5, 7, 8]. More commonly, a single injury is present in suicides by sharp force. However, there are also cases reporting multiple injuries, with a maximum of 92 injuries [3, 6, 9–12]. Usually, the dominant hand is used either alone or as the lead hand, with self-inflicted injuries present on the contralateral part of the body. The most commonly used implement is a knife, and it should be found somewhere near the body [4, 13, 14]. The etiology of suicidal behavior is complex and multifactorial. Among the most common risk factors is a psychiatric disorder [15].

Self-mutilation is defined as the deliberate self-destruction of a part of a person’s own body without conscious suicidal intent. Minor forms of self-mutilation, such as skin-cutting or nail-biting, are frequently encountered. On the other hand, acts of major self-mutilation are quite rare. Cases of eye, ear, tongue, limb, or genital self-mutilations have been reported [16–18]. This pattern of behavior is predominantly associated with psychotic illness and substance abuse. Previous self-mutilation actions, suicidal attempts, command hallucinations, and active symptoms associated with untreated schizophrenia are among the predictors of self-injurious behavior [18, 19]. Self-cannibalism is an extremely rare form of major self-mutilation [20, 21]. Although there have been reports on self-cannibalism after self-mutilation in psychosis or its absence, none has reported a fatal case where this behavior was associated with multiple self-inflicted stab and cut wounds [17, 20, 22–24].

The presented case was a suicide by sharp force with a few atypical features, including the number, location, and kind of self-inflicted sharp force injuries, the absence of a relationship between handedness and wound location (hesitation marks on both elbows, superficial cuts on both hands), and the absence of clothing. Also unusual was the setting of the suicide (the forest). The suicidal actions, most probably resulting from psychotic symptoms, were associated with self-amputation of parts of both ears, the fifth toe of the right foot, and the scrotum, followed by self-cannibalism.
